# Illumina sequencing analysis of the ruminal microbiota in high-yield and low-yield lactating dairy cows

**DOI:** 10.1371/journal.pone.0198225

**Published:** 2018-11-13

**Authors:** Jinjin Tong, Hua Zhang, Delian Yang, Yonghong Zhang, Benhai Xiong, Linshu Jiang

**Affiliations:** 1 Beijing Key Laboratory for Dairy Cow Nutrition, Beijing University of Agriculture, Beijing, P. R. China; 2 State Key Laboratory of Animal Nutrition, Institute of Animal Science, Chinese Academy of Agricultural Sciences, Beijing, P. R. China; University of Illinois, UNITED STATES

## Abstract

In this study, differences in the ruminal bacterial community between high-yield and low-yield lactating dairy cows under the same dietary conditions were investigated. Sixteen lactating dairy cows with similar parity and days in milk were divided into high-yield (HY) and low-yield (LY) groups based on their milk yield. On day 21, rumen content samples were collected, and their microbiota compositions were determined using high-throughput sequencing of the 16S rRNA gene by the Illumina MiSeq platform. During the study period, dry matter intake (DMI) and milk yield were measured daily, and milk composition was assessed 3 times per week. The results showed that the milk of the LY group tended to have higher fat (*P* = 0.08), protein (*P* = 0.01) and total solid contents (*P* = 0.04) than that of the HY group, while the HY group had higher ruminal propionate (*P* = 0.08) proportion and volatile fatty acid (VFA) (*P* = 0.02) concentrations. Principal coordinate analysis indicated significant differences in ruminal bacterial community compositions and structures between the HY group and LY group. The abundances of *Ruminococcus 2*, *Lachnospiraceae* and *Eubacterium coprostanoligenes* were significantly higher in the HY group than in the LY group. In addition, *Bacteroides*, *Ruminococcus 2* and *Candidatus-Saccharimonas* were positively correlated with ruminal propionate proportion (r>0.4, *P*<0.05). These findings enhance the understanding of bacterial synthesis within the rumen and reveal an important mechanism underlying differences in milk production in dairy cows.

## Introduction

A symbiotic relationship exists with regard to the rumen microbiota of cattle. The rumen is a highly specialized organ of ruminant animals that promotes a community of mutualistic microbial species while simultaneously absorbing nutrients derived from digestion of plant fibre and cellular material [1]. The rumen microbial community has a direct relationship with volatile fatty acid (VFA) and microbial protein biosynthesis, which play important roles in milk production efficiency [2]. In addition, the bacterial community also determines the production traits [3], production variables [4–7], and milk production and composition [8, 9] in dairy cows. Rumen microbial dynamics have been reported to involve both core and variable microbial components [10–12]. Similar to the microbial community in the gut of non-ruminants, the structure and function of the microbial community in the cow rumen are shaped by dynamic physical, chemical, and predatory environments [13, 14]. In turn, the microbial community regulates nutrient cycling to the host [15]. However, a more in-depth comparison is warranted to improve our understanding of differences in rumen bacterial community composition between high-yield and low-yield dairy cows.

Recent efforts to study the rumen microbiome have focused on identifying and quantifying ruminal microbial communities [8, 16]. As a powerful molecular approach for taxonomic analyses, the application of 16S rRNA gene sequencing technology has provided novel insight into the microbiome ecology of gastrointestinal tracts [17, 18]. Indeed, this technique has been widely used to study microbial diversity and the metabolic capabilities of microbiomes in different ecological niches [19], fermented food [20, 21], waste-water treatment facilities [22], and human and animal gastrointestinal tracts [23–25]. Recently, Paz et al. [26] reported on the compositions of various bacterial communities in different dairy breeds. Furthermore, some distinct rumen bacterial communities were significantly associated with the rumen fermentation parameters, which affect milk production [27]. Therefore, the objective of the present study was to examine differences in ruminal bacterial community compositions between high-yield and low-yield lactating cows under the same dietary conditions.

## Materials and methods

### Animals and experimental design

The experimental protocol was approved by the Institutional Animal Care and Use Committee at the Beijing University of Agriculture, in compliance with regulations for the administration of affairs concerning experimental animals (The State Science and Technology Commission of P. R. China, 1988). According to the principle of parity and lactation days, 16 Holstein lactating dairy cows of similar parity were used and assigned to a high-yield group (average production 31.90±1.76 kg/d, mean±SD) or a low-yield group (average production 19.30±1.76 kg/d), with 8 each. All the cows used in this experiment averaged 2.6±0.4 parity. At the beginning of the experiment, the average days in milk (DIM) was 114.6±7.5 days, the average body weight was 670±24 kg and the average dry mater intake was 24.2±2.7 kg/d. There was no initial difference between groups in terms of these parameters except the milk production. The cows had free access to water and were housed in a tie-stall barn. Cows were milked 3 times per day (0700,1400 and 2100 h). Feed intake and milk production data were recorded daily throughout the experiment. The experimental duration was 21 d, with an adaptation period of 14 d as control period and a sampling period of 7 d (D15-D21). These lactating dairy cows were the fed under the same dietary conditions, the composition of which is shown in Table 1.

**Table 1:** Ingredients and nutrient composition (% of DM) of the basal diet.

Item	Content
**Ingredient, % of DM**	
**Alfalfa hay**	6.90
**Corn silage**	46.32
**Oat grass**	2.40
**Ground corn**	9.88
**Soybean meal**	5.10
**Steam-flaked corn**	4.40
**DDGS**^1^	4.40
**Corn bran**	3.70
**Extruded soybean**	3.00
**Barley**	2.66
**Wheat barn**	2.66
**Sodium cyclamate**	2.40
**Oat**	1.50
**Canola meal**	1.07
**Cottonseed meal**	1.07
**Magalac**^2^	0.90
**NaHCO**_**3**_	0.59
**Limestone**	0.48
**NaCl**	0.27
**Premix**^3^	0.30
**Nutrient composition**^4^	
**CP**	17.4
**NDF**	31.1
**ADF**	16.6
**Ether extract**	5.00
**Ca**	0.78
**P**	0.44
**NE**_**L**_**, Mcal/kg**	1.76

^1^DDGS = dried distillers’ grain with solubles.

^2^Church and Dwight Co. Inc., Princeton, NJ.

^3^Formulated to provide (per kg of DM) 4,560 mg of Cu, 3,000 mg of Fe, 12,100 mg of Zn, 4,590 mg of Mn, 60 mg of Co, 200 mg of Se, 270 mg of I, 10,000 IU of vitamin E, 450,000 IU of vitamin D, 2,000,000 IU of vitamin A, and 3,000 mg of nicotinic acid.

^4^Chemical composition is based on chemical analysis of the total mixed ration (TMR), as described.

### Rumen fluid sampling and parameter measurement

Rumen fluid samples were collected from the oral cavity at 3–4 h after the morning feeding on day 21 (D21). The rumen contents were strained through 4 layers of cheesecloth with a mesh size of 250 μm. Ruminal pH was immediately measured using a portable pH metre (Testo 205, Testo AG, Germany). The filtered rumen fluid samples were centrifuged at 10,000 × g for 15 min at 4°C, aliquoted into 5-mL cryopreservation tubes, frozen in a liquid nitrogen tank and stored at -80°C until analysis of the ruminal bacterial community. A VFA analysis was conducted on 1 mL of each rumen fluid sample, which was preserved by adding 0.2 mL of 25% HPO_3_, by gas chromatography as reported by Mao et al. [28]. An ammonia-N analysis was performed using a colorimetric method consistent with AL and EP [29].

### DNA extraction and polymerase chain reaction (PCR) amplification

Microbial DNA was extracted from rumen fluid samples using EZNA Bacterial DNA Kit (Omega Bio-Tek, Norcross, U.S.) according to the manufacturer’s protocols.

The yield and purity of the extracted DNA were assessed with a NanoDrop 1000 instrument (NanoDrop, Wilmington, DE).

### 16S rRNA analysis

The V3-V4 regions of the 16S ribosomal RNA gene were amplified by PCR. The reaction mixture contained 4 μL of 5 × FastPfu Buffer, 2 μL of 2.5 mM deoxyribonucleotide triphosphates (dNTPs), 0.8 μL of each primer (5 μM), 0.4 μL of FastPfu Polymerase, 0.2 μL of bovine serum albumin (BSA) and 10 ng of the template DNA. The PCR protocol was set as follows: 95°C for 3 min, followed by 27 cycles at 95°C for 30 s, 55°C for 30 s, 72°C for 45 s and a final extension at 72°C for 10 min. The sequences of the primers used for PCR were as follows: 338F 5’-barcode-ACTCCTACGGGAGGCAGCAG)-3’ and 806R 5’- GGACTACHVGGGTWTCTAAT-3’. The reactions were performed in triplicate on 20-μL mixtures. Amplicons were excised from 2% agarose gels and purified using AxyPrep DNA Gel Extraction Kit (Axygen Biosciences, Union City, U.S.) according to the manufacturer’s instructions and then quantified using a NanoDrop 2000 (Thermo, U.S.). Purified amplicons were pooled in equimolar ratios and pair-end sequenced (2 × 300) on the Illumina MiSeq platform according to standard protocols. The raw reads were deposited into the NCBI Sequence Read Archive (SRA) database (Accession Number: SRP136923).

### Statistical analysis

Data of dry matter intake, milk yield, milk composition, ruminal pH, VFA concentrations, and alpha diversity index were analyzed using PROC MIXED of SAS 9.4 (SAS Institute, Inc, Cary, NC) as shown in the following model: Yij = μ + Ti + ej, where Yij is the dependent variable, μ is the overall mean, Ti is the effect of treatment (LY or HY, considered fixed), and ej is the residual. A *P*<0.05 was considered statistically significant, and a trend was indicated by *P*<0.10. A principal coordinate analysis (PCoA) and Pearson correlations were carried out using R-3.2 with vegan package on the online Majorbio I-Sanger Cloud Platform (http://www.i-sanger.com). Pearson correlations were used to analyse the environmental factors and bacterial relationships using the pheatmap package. The resulting numerical matrix is visually displayed in a heat map diagram. The colour change reflects the data information in a two-dimensional matrix or table. The colour depth indicates the size of the data value (correlation value). The matrix directly shows the size of the data value with the defined colour. Asterisks indicate that the correlation coefficients (r) were >0.4 and the *P* values were <0.05.

## Results

### Dry matter intake, milk yield, and milk composition

Dry matter intake (DMI) was significantly greater in the high-yield (HY) group than in the low-yield (LY) group (*P* = 0.03). Milk production, 4% fat-corrected milk (FCM) and energy-corrected milk (ECM) were significantly lower in the LY group than in the HY group (Table 2). The milk fat content tended to be higher (*P* = 0.08), and the milk protein content was significantly higher in the LY group than in the HY group (*P<*0.01). No difference was observed in milk lactose content between the LY and HY groups (*P* = 0.21). Fat, protein and lactose yields were significantly greater in the HY group than in the LY group. However, somatic cell count (SCC) was not different between the HY and LY groups (*P* = 0.13; Table 2).

**Table 2:** Milk and ECM from high-yielding and low-yielding dairy cows during the entire sampling period^1^.

Items	LY	HY	SEM	*P*-value
**DMI (kg/d)**	23.4	25.6	0.43	0.0347
**Milk production (kg/d)**	19.3	31.9	1.76	<0.0001
**4%FCM production (kg/d)**^**2**^	18.95	29.20	1.73	<0.0001
**ECM production (kg/d)**^**3**^	21.07	32.09	1.93	<0.0001
**Milk composition**				
**Fat %**	4.02	3.48	0.28	0.0788
**Fat yield (kg/d)**	0.75	1.10	0.07	0.0004
**Protein %**	3.50	3.07	0.11	0.0023
**Protein yield (kg/d)**	0.66	0.98	0.06	0.0002
**Lactose %**	4.87	5.05	0.14	0.2127
**Lactose yield (kg/d)**	0.92	1.61	0.10	<0.0001
**Total solid content %**	13.15	12.30	0.38	0.0417
**SCC (log**_**10**_**)**	2.46	3.10	2.78	0.1339

^1^Data are presented as least squares means.

SCC = somatic cell count.

### Ruminal pH and VFA concentrations

Rumen pH was not different between the HY group and the LY group (Table 3). NH_3_-N (mg/dL) was greater in the HY group than in the LY group (*P*<0.01), and there was an increase trend proportion of propionate (*P* = 0.08) and total VFA concentrations in the HY group relative to the LY group (*P*<0.05). In addition, the proportion of acetate had a tendency lower in the HY group than in the LY group (*P* = 0.06). A lower trend was observed for the acetate to propionate ratio in the HY group compared to the LY group (*P* = 0.06).

**Table 3:** Effects of differences between high-yielding and low-yielding dairy cows on metabolites in the rumen.

Items	LY	HY	SEM	*P*-value
**pH**	6.73	6.71	0.02	0.69
**NH**_**3**_**-N, mg/dL**	7.99	13.28*	1.03	0.01
**Proportion**				
**Acetate**	62.38	60.72	0.45	0.06
**Propionate**	21.95	23.14	0.34	0.08
**Isobutyrate**	0.83	0.74	0.03	0.13
**Butyrate**	12.06	12.55	0.22	0.27
**Isovalerate**	1.21	1.31	0.05	0.34
**Valerate**	1.58	1.55	0.04	0.74
**Acetate:propionate ratio**	2.85	2.64	0.06	0.06
**Total VFA (mmol/L)**	99.76	113.63*	3.14	0.02

*P<0. 05

Values within a sampling day followed by superscripted asterisks differ.

SEM = standard error of the mean.

### Diversity and richness of microbial communities

In total, 2,382,338 merged sequences were acquired for the 16 samples from the dairy cows and 1,191,169 high-quality sequences, with an average read length of 440 bp, were classified as bacterial. On average, at least 54,144 sequences were obtained per sample, and greater than 99% depth coverage was achieved. The rarefaction curve generated tended to plateau, showing that the number of OTUs did not rise with an increasing volume of data. This finding showed that the data volume of sequencing was reasonable. The results of this study show that the sequencing data were reasonable and could reflect changes in most bacterial flora.

No significant differences were observed in alpha diversity index results between the HY and LY groups (*P*>0.05) (Table 4). However, the coverage of the HY group was significantly higher than that of the LY group (*P*<0.01), indicating greater community diversity in the HY group.

**Table 4:** Alpha diversity index of rumen bacteria.

Item	LY	HY	SEM	*P*-value
**Sobs**	1196.13	1214.88	17.21	0.60
**Shannon**	5.41	5.49	0.04	0.42
**Simpson**	0.01	0.01	0.00	0.74
**ACE**	1375.65	1360.65	18.75	0.70
**Chao**	1378.64	1376.82	20.10	0.97
**Coverage**	0.992	0.994**	0.00	0.001

*P<0. 05

**P<0. 01:

Values within a sampling day followed by superscript asterisks differ. SEM = standard error of the mean

**Fig 1:**
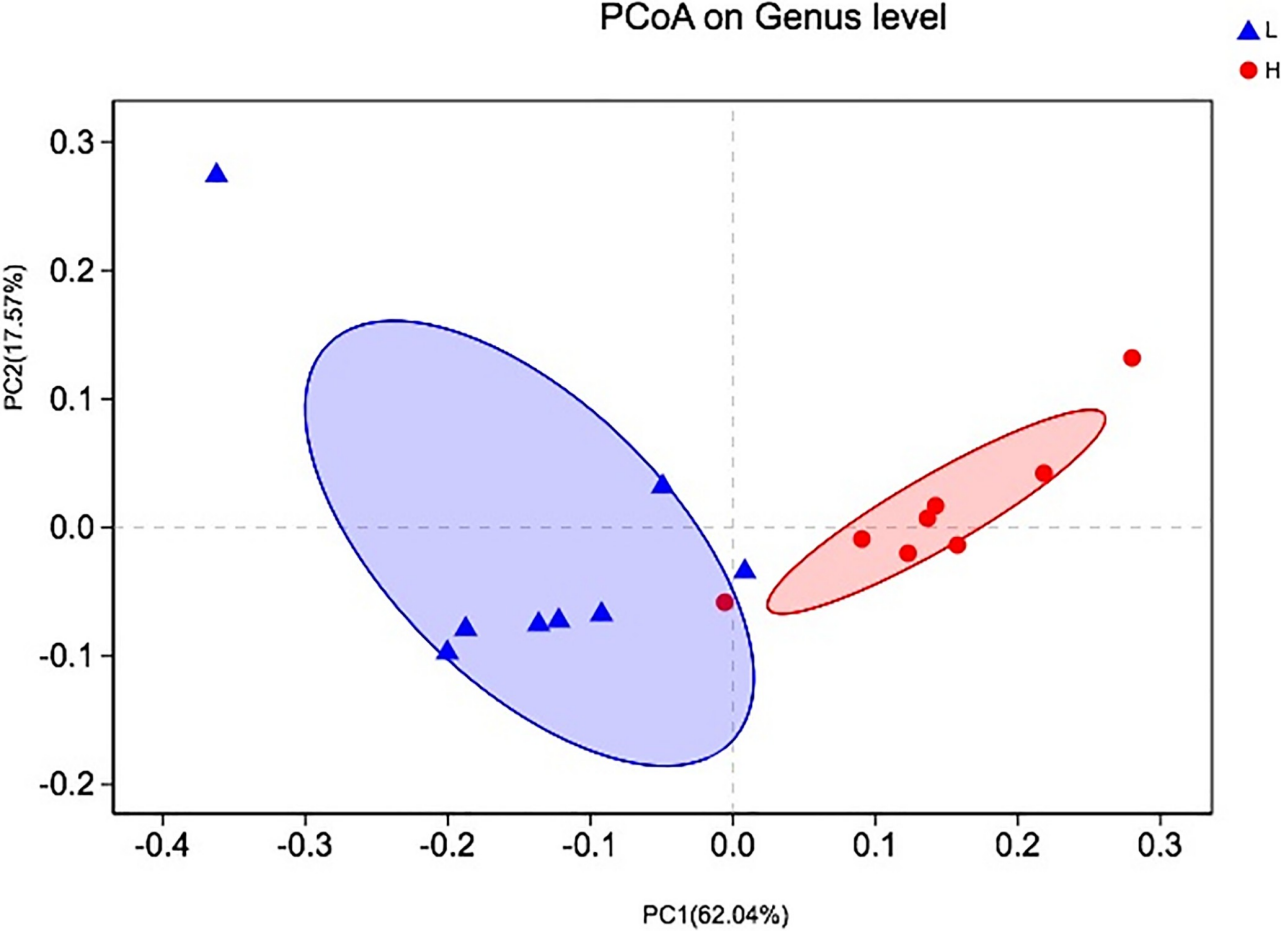
Principal coordinate analysis (PCoA) of bacterial community structures of the ruminal microbiota in the high-yielding group (red circles) and the low-yielding group (blue triangles). PCoA plots were constructed using the unweighted UniFrac method.

To understand the differences in the overall rumen bacterial community between high- and low-yield lactating dairy cows, a PCoA was used to analyse bacterial diversity, followed by the weighted UniFrac metrics (Fig 1). As shown in Fig 1, principal coordinate 1 accounted for 62.04% and principal coordinate 2 accounted for 17.57% of the total variation.

**Fig 2:**
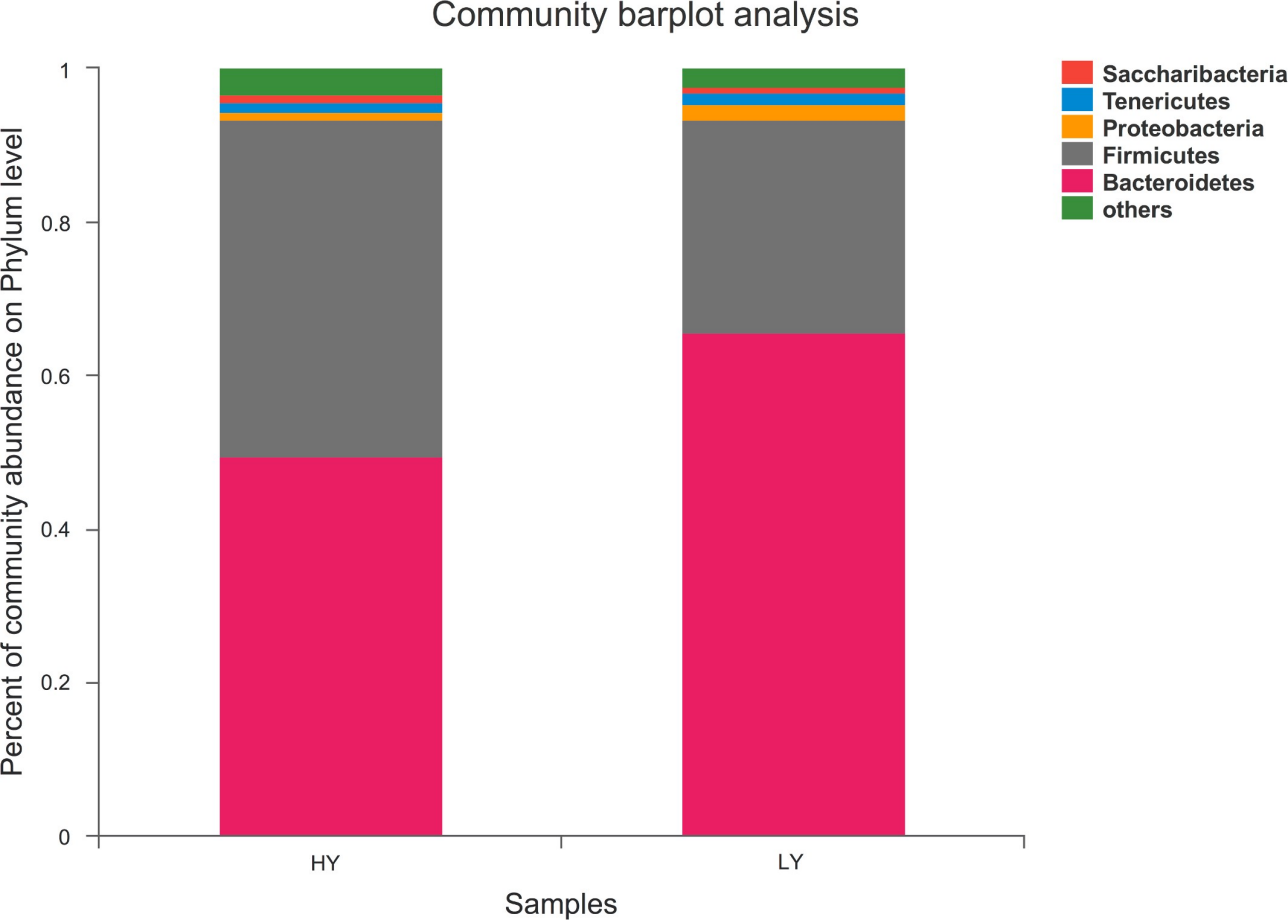
Percent composition of predominant phyla in the rumen fluid.

Twenty-one bacterial phyla were identified across all samples. Bacteroidetes, Firmicutes and Proteobacteria were the three dominant groups, representing 57.59%, 35.86%, and 1.53% of the total sequences, respectively (Fig 2). Thus, at the phylum level, Bacteroidetes and Firmicutes were particularly dominant. The HY group exhibited a greater abundance of Firmicutes and lower abundance of Bacteroidetes than did the LY group (*P*<0.01), whereas Proteobacteria was less abundant (*P*<0.05) (S1 Table).

**Fig 3:**
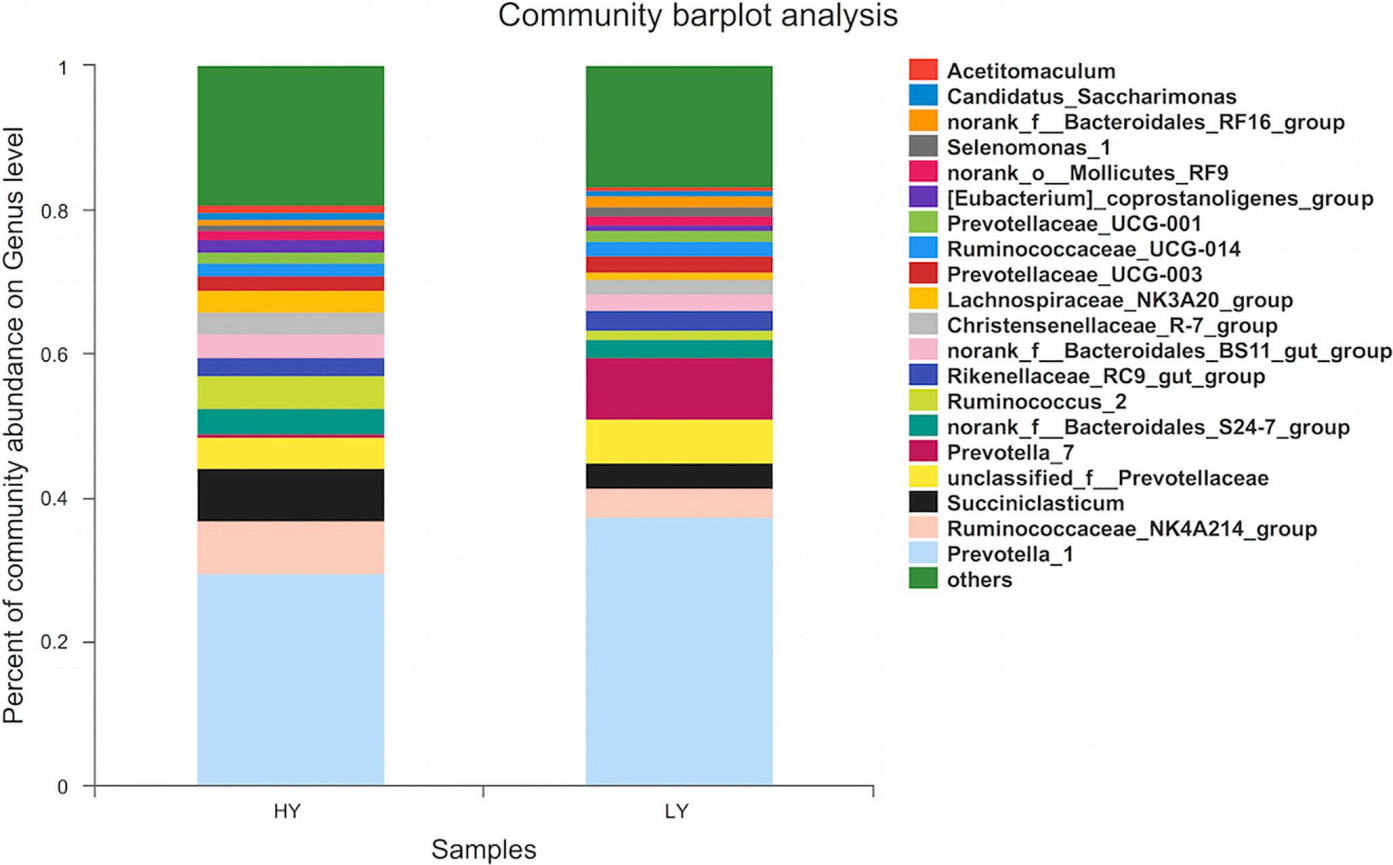
Percent composition of genera in the rumen fluid.

**Fig 4:**
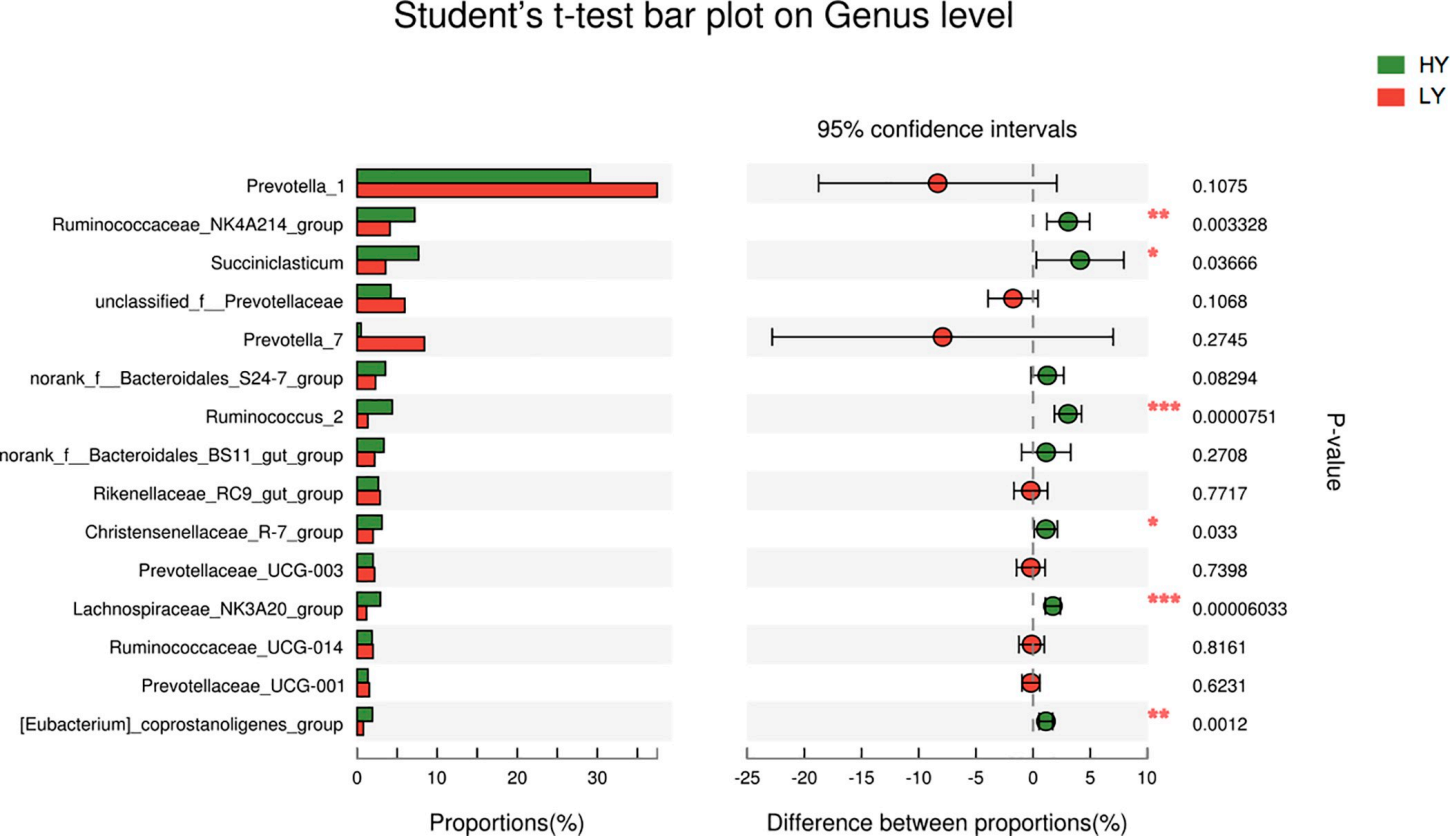
Percent composition and significance of genera in the rumen fluid.

At the genus level, taxa with a relative abundance of ≥1% in at least one sample were further analysed, and the relevant genera are presented in Figs 3 and 4. Twenty-one genera were identified, 6 of which exhibited significantly different abundances between the groups. Specifically, 4 genera were more abundant in the HY group at *P*<0.01, including *Ruminococcaceae*-*NK4A214-group*, *Ruminococcus 2*, *Lachnospiraceae-BS11-gut-group*, and *[Eubacterium]-coprostanoligenes-group*, and 2 were more abundant in the HY group at *P*<0.05: *Succiniclasticum* and *Christensenellaceae-R-7-group* (S1 Table).

### Correlations between bacterial communities and ruminal variables

**Fig 5:**
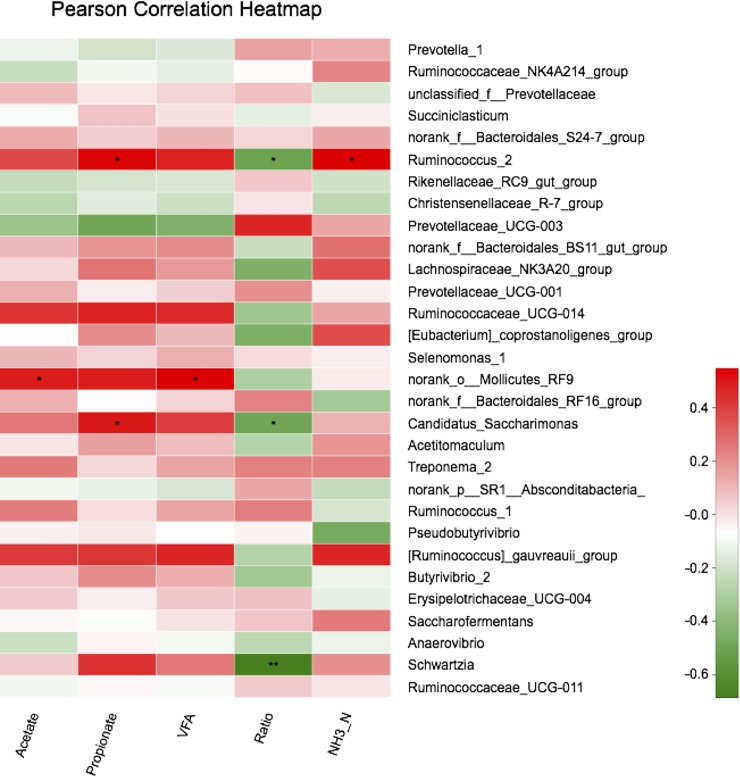
Correlation analyses between the relative abundances of bacteria genera and ruminal fermentation parameters. Only genera with abundances significantly associated with ruminal VFA, propionate and acetate concentrations are presented. Green represents a negative correlation between the abundance of the species and the ratio (r<−0.4), and red represents a positive correlation (r>0.4, 0.01<*P*< = 0.05 *; 0.001<*P*≤0.01 **; *P*≤0.001 ***; Values with a significant correlation followed by superscripted asterisks differ).

As shown in Fig 5, the relative abundances of the genera *Bacteroides* and *Ruminococcus 2* were positively correlated with ruminal propionate and NH_3_-N concentrations (r>0.4, *P*<0.05) but negatively correlated with the ruminal ratio (acetate:propionate ratio) (r<-0.4, *P*<0.05). In addition, *norank_o__Mollicutes_RF9* was positively correlated with ruminal acetate and VFA concentrations (r>0.4, *P*<0.05). *Candidatus-Saccharimonas* was positively correlated with the ruminal propionate concentration (r>0.4, *P*<0.05) but negatively correlated with the ruminal ratio (r<-0.4, *P*<0.05). Moreover, the ratio was negatively correlated with *Schwartzia* (r<-0.6, *P*<0.05).

## Discussion

### Changes in rumen fermentation parameters and milk composition

Higher volatile fatty acid concentration and milk composition yield were found in the HY group compared to the LY group. Recent studies suggested an important relationship between VFAs and milk components [2, 30, 31]. Specifically, of the three principle VFAs, acetate and butyrate are substrates for oxidation and are precursors of lipids [32, 33], moreover propionate is the only glucogenic VFA, accounting for 65–80% of the net glucose supply in lactating dairy cows [34, 35]. In the present study, the proportion of propionate had an increasing trend in the HY group than that in the LY group, which may also be explained the mechanism of different milk composition and milk production. In line with the reported that milk yield was most highly related to rumen concentrations of butyrate and propionate [36].

Milk fat, protein and lactose yields were significantly greater in the HY group than in the LY group, which is consistent with previous research that milk production and VFA-producing bacterium have the positive correlation[37]. Weimer et al. [14] also showed that high-efficiency Holstein cows have greater VFA and propionate molar percentages compared with low-efficiency Holstein cows, which was apparently caused by differences in the ruminal bacterial community. Furthermore, it has been reported that even under the same dietary condition the bacterial communities also different between Holstein and Jersey cows [26]. Therefore, these findings suggested that probably due to host–microbiota interactions, different milk production dairy cows may harbour different microbial species compositions, which are probably closely related to distinct differences in rumen fermentation parameters and milk composition.

The NH_3_-N concentration in rumen fluid can reflect the balance of protein degradation and synthesis under varying feed conditions. Our results showed that the NH_3_-N concentration was within the normal range, though that in the HY group was significantly higher than that in the LY group. It is well known that NH_3_-N is an intermediate product of feed protein, non-protein nitrogen degradation and microbial protein synthesis, and it is mainly affected by feed protein degradation, rumen wall absorption, microorganism utilization and rumen chyme outflow rate [38–40]. Yang et al. [41] reported that the concentration of NH_3_-N should be higher than 5 mg/dL; otherwise, it will influence the "uncoupling" effect of ruminal fermentation and reduce the efficiency of microbial protein synthesis. Corroborating all these results indicate that rumen microbes promote protein degradation in high-yield dairy cows, providing a better understanding of the difference in milk proteins between the two groups.

### Differences in rumen microbial composition between HY and LY groups

No differences were observed in bacterial community richness and diversity between the groups in our study. Three phyla predominated in both groups, which was consistent with previous studies reporting that the principal phyla of microbes in the rumen are Bacteroidetes, Firmicutes, and Proteobacteria [38]. The proportions of these three phyla account for approximately 94% of the total [40, 42, 43]. Interestingly, our results showed that the abundance of Firmicutes in the HY group was higher than that in the LY group, though the abundances of the two other dominant phyla were lower in the former than in the latter. In consistent with previous reported by Pan et al. [44], cows fed a high proportion of grain have a higher abundance of Firmicutes and a lower abundance of Proteobacteria than control cows, and other studies have shown that feeding a high amount of grain can promote milk production [45, 46]. Thus, our study provides a better understanding of why cows fed the same dietary condition can have different milk production. The present findings further demonstrate that Firmicutes plays an important role in milk production.

In agreement with other research results [12, 47], our study showed that *Prevotella* was the most abundant genus in all samples. Although *Prevotella* was more abundant in the LY group than in the HY group, the difference was not significant. In contrast, *Ruminococcaceae*-*NK4A214-group*, *Ruminococcus 2*, *Lachnospiraceae-BS11-gut-group* and *[Eubacterium]-coprostanoligenes-group* were significantly different between the two groups, with higher abundances in the HY group than in the LY group. Jiang et al. [48] reported that the increase in the relative abundance of *Ruminococcus* partly explains why adding live yeast to the diet increases the in vivo digestibility of DM and NDF and the performance of cows. Thus, this result illustrates that high-performance cows have higher abundances of *Ruminococcus* in the rumen fluid, which is consistent with the present research results. Besides, *Ruminococcus* spp. as major bacteria plays an important role in acetate production [49]. However, our results found that although the *Ruminococcus* spp. had significantly higher abundances in the HY group than in the LY group, the proportion of acetate was trend to lower in HY group compared with LY group.

Members of the family *Lachnospiraceae* are gram-positive obligate anaerobes that are mostly non-spore-forming bacteria [50, 51]. Huws et al. [52] showed that *Ruminococcaceae* and *Lachnospiraceae* play predominant roles in biohydrogenation pathways within the rumen. Furthermore, as the primary succinate-utilizing bacterial taxon, *Succiniclasticum* accounted for 7.45% of the total bacterial community in the HY group, with significantly greater abundance than in the LY group. A higher level of *Succiniclasticum* has been associated with greater production of succinate from starch degradation [53], which could also explain the higher proportion of propionate in HY group compared with LY group. Moreover, the abundances of *Christensenellaceae* and *Ruminococcaceae NK4A214* in the HY group were significantly higher than in the LY group, though little information about these two genera has been reported in the literature. The reasons for the altered status of genera in cows with different milk production are need further studies.

## Conclusion

In summary, high-yield dairy cows have better ruminal fermentation patterns than do low-yield cows, which was partially attributed to the greater abundances of *Bacteroides*, *Ruminococcus 2*, *Ruminococcaceae NK4A214*, *Lachnospiraceae*, *Succiniclasticum*, *Eubacterium* and *Christensenella* in the former. Furthermore, rumen fermentation in high-yield cows exhibited higher VFA levels than that found in low-yield cows. The rumen microbial compositions of high-yield and low-yield dairy cows are different, and microbial species diversity and distribution contribute to production-related phenotypes. Overall, our findings enhance our understanding of rumen bacteria in cows with different milk yields and provide new strategies for improving dairy cow production performance.

## Supporting information

S1 Table Differences rumen microbial abundances between high-yielding and low-yielding dairy cows. (XLSX)

S2 Table Sequence analysis raw data_otu_taxon. (XLS)
